# Chronic appendicitis: two case reports 

**DOI:** 10.1186/s13256-022-03273-2

**Published:** 2022-02-09

**Authors:** Nanna Holm, Maria Unni Rømer, Elena Markova, Laura Katrine Buskov, Ann-Brit Eg Hansen, Michala Vaaben Rose

**Affiliations:** 1 Department of Infectious Diseases, Copenhagen University Hospital Hvidovre, Kettegaard Allé 30, 2650 Hvidovre, Denmark; 2 Department of Pathology, Rigshospitalet, Copenhagen University Hospital, Blegdamsvej 9, 2100 Copenhagen, Denmark; 3 Department of Clinical Physiology and Nuclear Medicine, Copenhagen University Hospital Hvidovre, Kettegaard Allé 30, 2650 Hvidovre, Denmark; 4 Department of Radiology, Copenhagen University Hospital Hvidovre, Kettegaards Allé 30, 2650 Hvidovre, Denmark

**Keywords:** Case report, Chronic appendicitis, Recurrent abdominal pain, Radiologic imaging

## Abstract

**Background:**

Chronic appendicitis is a condition unfamiliar to many physicians and is often referred to as a controversial diagnosis. This can give rise to diagnostic delay.

**Case presentation:**

We present two cases of chronic appendicitis: a Caucasian female aged 21 years and a Caucasian male aged 34 years. The patients had different clinical presentations, which led the initial investigations in very different directions—tropical infectious disease and possible malignancy, respectively. In both cases, radiological imaging was the key investigation leading to the final surprising diagnosis.

**Conclusion:**

With these two case stories, we wish to draw attention to chronic appendicitis as a possible differential diagnosis in younger patients with chronic or recurrent abdominal pain, particularly if the pain is located in the lower abdomen and is accompanied by fever.

## Background

In general practice, patients frequently present with abdominal pain, with a high prevalence of acute underlying disease [1]. Acute appendicitis is among the common differential diagnoses, with an estimated lifetime risk of 7–8% [2]. Chronic appendicitis has often been referred to as a controversial diagnosis and its prevalence is unknown.

We present two cases of chronic appendicitis where the patient presentation led the investigations in very different directions, thus delaying the diagnosis.

## Case presentation

### Patient 1

**Fig. 1.**
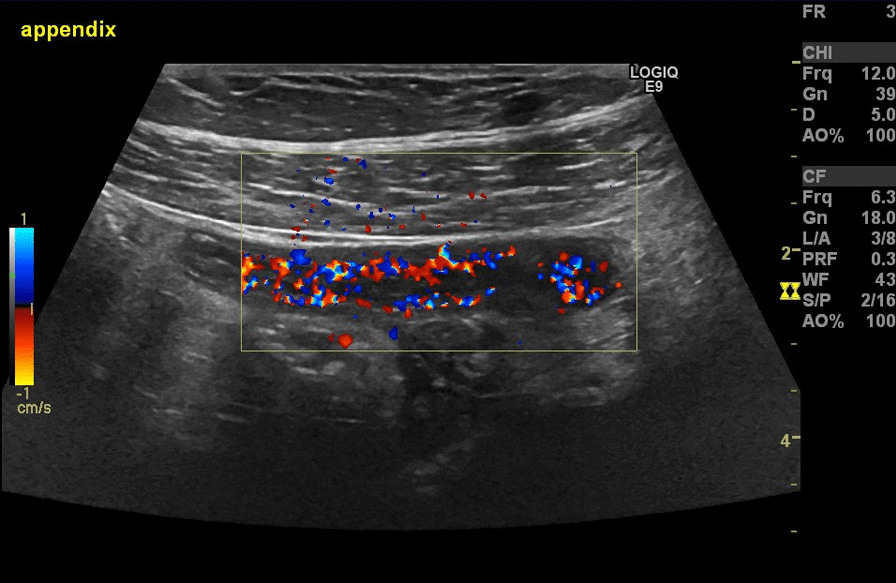
Patient 1. Ultrasound of lower right quadrant. Ultrasound of the right lower quadrant (area of maximum tenderness) displaying layering and thickening (10 mm) of the distal appendix with increased vascularity and moderate periappendicular edema. No abscess or signs of perforation

A 21-year-old Caucasian woman, previously healthy and asymptomatic with no family history of abdominal disease, presented with 2 months of recurrent, dull abdominal pain in the upper part of her abdomen with no radiation that lasted for 1–2 days approximately twice a week. The pain was often accompanied by fever and 2–3 times by nausea and vomiting and was not associated with defecation or urination. There were no known aggravating or alleviating factors of the abdominal pain. The symptoms initially began with 1 week of gastroenteritis starting the day of her return from a backpacking holiday to the Maldives, Sri Lanka, Bali, and Singapore. After the first week, she had no recurrent episodes of diarrhea and no weight loss, but continued with recurrent episodes of abdominal pain and fever. She had no complaints of abdominal pain prior to her holiday. On examination, we found distinct abdominal tenderness in the right lower quadrant with no palpable tumors. She had a leukocyte count of 7.9 × 10^9^/L (normal range: 3.5–8.8 × 10^9^/L), C-reactive protein (CRP) 37 mg/L (normal range: < 10 mg/L), and normal hemoglobin and liver-parameters. Urine human chorionic gonadotropin (U-hCG) was negative and fecal calprotectin < 30 × 10^−6^ (normal range: < 50 × 10^−6^). Given the initial suspicion of traveler’s diarrhea or another tropical infectious disease, several microbiological examinations were undertaken, including malaria testing × 3, blood cultures; fecal cultivation and polymerase chain reaction (PCR) for pathogenic bacteria, virus, and parasites (Table 1); fecal samples for analyses of intestinal worms, eggs, and cysts; and tests for human immunodeficiency virus (HIV), hepatitis A/B/C, cytomegalovirus, and Epstein–Barr virus. All tests were negative. An ultrasonic scan was performed, displaying a thickened, hypoechoic, and hyperemic appendix with edema of the surrounding fat (Fig. 1). Due to the patient history, the diagnosis of chronic appendicitis was suggested, but as the findings were considered controversial, an additional computed tomography (CT) scan was performed confirming the suspicion of chronic appendicitis with segmental thickening and increased contrast uptake of the appendix. No antibiotics were prescribed. Elective surgery was planned, but due to worsening of the abdominal pain the patient underwent acute surgery 1.5 months after the first hospital visit, hence 3.5 months after her symptoms began.

**Table 1:** Fecal examination for bacteria (cultivation and PCR), viruses (PCR), and parasites (PCR)

Bacteria	Viruses	Parasites
*Salmonella* species	Adenovirus	*Giardia lamblia*
*Shigella* species	Rotavirus	*Entamoeba histolytica*
*Yersinia*	Norovirus genotype I, II	*Cryptosporidium*
*Campylobacter*	Sapovirus	
*Aeromonas*		
*Escherichia coli*^a^		

^a^*E. coli* species tested for Verocytotoxin-producing *E. coli* (VTEC), enteropathogenic *E. coli* (EPEC), enterotoxigenic *E. coli *(ETEC), and enteroinvasive E. coli (EIEC) of intimin-producing *E. coli*

The pathological examination of the appendix showed signs of previous perforation, transmural chronic inflammation including fibrosis, and non-necrotizing granulomas. Testing for tuberculosis and fungal and specific microorganisms (periodic acid-Schiff stain for fungal organisms and parasites) were negative.

Eighteen months after the appendectomy, the patient was symptom-free.

### Patient 2

**Fig. 2:**
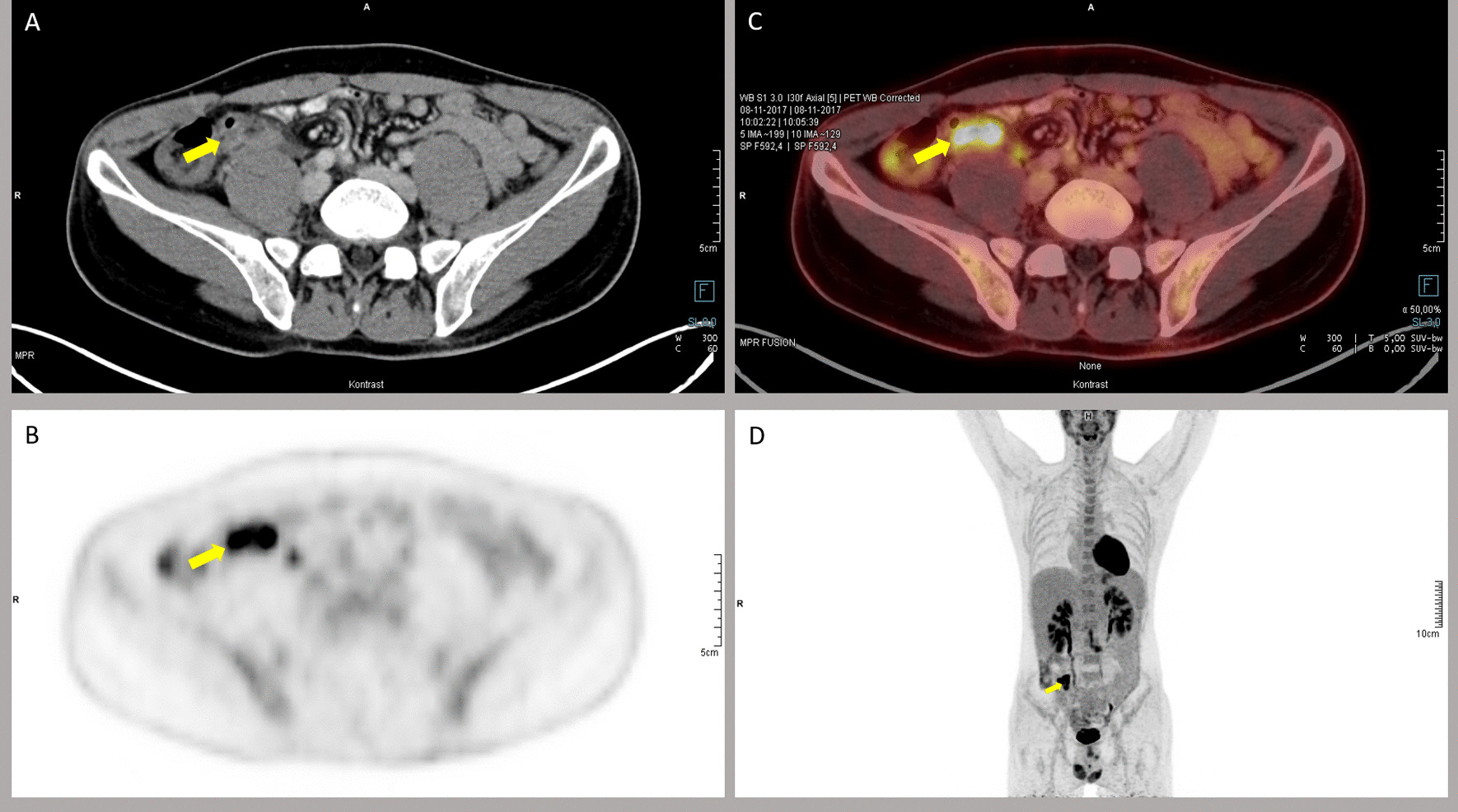
Patient 2. ^18^F-FDG PET/CT. **A** CT-scan with intravenous and per oral contrast (transaxial plane) of the abdomen at the level of the appendix showing increased diameter of the appendix. **B** ^18^F-FDG PET (transaxial at the same level as the CT scan) coincides with the increased diameter of the appendix observed on the CT scan. **C** Combined ^18^F-FDG PET/CT showing the increased signal in the appendix corresponding to inflammation of the appendix. **D** Maximum intensity projection (MIP)

A 34-year-old Caucasian man, previously healthy with no family history of abdominal disease, was referred to the specialist fast-track department to rule out possible malignancy due to unintended weight loss of 8 kg during the past 9 months despite no changes in his usual diet. In the same time period he had experienced four episodes, lasting approximately 1 week, of fever and lower abdominal pain, accompanied by vomiting on the first day. The episodes were self-limiting without any antibiotic treatment. On examination, the patient had abdominal tenderness in the left lower quadrant. Hemoglobin level was 8.5 mmol/L, leukocyte count of 7.2 × 10^9^/L, CRP 160 mg/L, and D-dimer 1.4 U/L (normal range: < 0.5 U/L). Other blood results included thrombocytes, liver enzymes, creatinine, thyroid stimulating hormone, hemoglobin A1c, M-component, and antinuclear antibodies—all normal. Fecal calprotectin was 37 × 10^−6^, no fecal occult blood testing was performed. HIV testing and fecal cultivation and PCR for pathogenic bacteria were negative (Table 1). Blood cultures became positive after 1 day of incubation with growth of *Bacteroides fragilis* and *Streptococcus intermedius*. ^18^F-fluoro-2-deoxy-d-glucose positron emission tomography/computed tomography (^18^F-FDG PET/CT) showed increased diameter and signal of the appendix corresponding to inflammation of the appendix and thrombosis in the superior mesenteric veins (Fig. 2). Colonoscopy was normal.

The patient was treated with antibiotics for 7 days (meropenem 1 g intravenously 3 times daily for 3 days followed by moxifloxacin 400 mg orally for 4 days, in conjunction with metronidazole 500 mg orally 3 times daily for the full 7 days ) and the symptoms resolved. The thrombosis was treated with warfarin for 6 months. ^18^F-FDG PET/CT 1 month later showed regression of the previous findings and hence appendectomy was not undertaken.

Nineteen months later, during which time he had been symptom free, the patient was admitted with acute abdominal pain with localized rebound tenderness on palpation in the right lower quadrant. He was afebrile, with a leukocyte count of 11.9 × 10^9^/L and CRP of 18 mg/L. No radiological imaging was undertaken. Laparoscopic appendectomy revealed a phlegmonous, gangrenous appendix with adherences to the adjacent terminal ileum mesentery. The pathology report described an ulcerated surface with acute transmural inflammation, no granuloma, and no malignancy.

Eight months after the appendectomy the patient was symptom-free.

## Discussion

Chronic appendicitis is a diagnosis unfamiliar to many clinicians, and with no official diagnostic criteria. A symptom duration of > 7 days of chronic or recurrent abdominal pain has previously been suggested to distinguish between acute and chronic appendicitis [3]. The symptoms are often milder than in acute appendicitis, which can lead to misdiagnosis and diagnostic delay.

Acute appendicitis can be caused by luminal obstruction or infection. Genetic factors and environmental influences may also be of importance in the development of appendicitis [2]. The etiology of chronic appendicitis is unknown but is likewise believed to be a result of partial or transient obstruction of the appendiceal lumen [4]. It is unknown whether chronic appendicitis is always preceded by an untreated or insufficiently treated acute appendicitis, or if chronic appendicitis is an independent disease entity. In our first case, gastroenteritis was possibly the initial cause of the chronic appendicitis, however, the travel history contributed to the delay in diagnosis as a tropical disease was suspected. In the second case, suspicion of malignancy led to the ^18^F-FDG PET/CT scan, which diagnosed appendicitis, as well as mesenteric thrombosis. The recurrent episodes of fever and lower abdominal pain, during the 9 months preceding diagnosis were all self-limiting and therefore not considered characteristic of acute appendicitis.

The radiological findings by CT in chronic appendicitis have been estimated to be identical to the findings in acute appendicitis and include pericecal stranding, dilation of appendix, apical thickening, and adenopathy [5].

In several cases, pathological findings, rather than the clinical presentation, have led to the final diagnosis. The pathological findings of chronic appendicitis include infiltration by lymphocytes, histiocytes, and plasma cells in lamina propria; hyperplasia of lymphoid tissue; and fibrosis [3].

In two retrospective studies, including 269 and 322 patients with appendicitis, histological signs of chronic appendicitis were found in 14% and 23% of the cases, respectively [3, 6]. In addition, these studies showed that 82–93% of the patients with chronic appendicitis became symptom-free after surgery. Antibiotic treatment is efficient in cases of acute appendicitis [7], but to our knowledge there are no clinical studies of antibiotic treatment in chronic appendicitis, hence optimal treatment strategy for this condition is unknown.

## Conclusion

With these two case stories, we wish to draw attention to chronic appendicitis as a possible differential diagnosis in younger patients with chronic or recurrent abdominal pain, particularly if the pain is located to the lower abdomen and is accompanied by fever. Radiological imaging with ultrasound and/or CT scan can be useful.

## Data Availability

All data analyzed during this study are included in this published article.

## Abbreviations

L: Liters
g: Grams
mg: Milligrams
CRP: C-reactive protein
U-hCG: Urine human chorionic gonadotropin
PCR: Polymerase chain reaction
HIV: Human immunodeficiency virus
CT: Computed tomography
^18^F-FDG PET/CT: ^18^F-fluoro-2-deoxy-d-glucose positron emission tomography/computed tomography
